# Molecular evolution of hatching enzymes and their paralogous genes in vertebrates

**DOI:** 10.1186/s12862-022-01966-2

**Published:** 2022-02-02

**Authors:** Tatsuki Nagasawa, Mari Kawaguchi, Kohki Nishi, Shigeki Yasumasu

**Affiliations:** 1 School of Life Science and Technology, Tokyo Institute of Technology, 2-12-1 Ookayama, Meguro-ku, Tokyo, 152-8550 Japan; 2 Department of Materials and Life Sciences, Faculty of Science and Technology, Sophia University, 7-1 Kioi-cho, Chiyoda-ku, Tokyo, 102-8554 Japan

**Keywords:** Hatching enzyme, C6astacin, Molecular evolution, Teleosts, Gene duplication, Neo-functionalization

## Abstract

**Background:**

Hatching is identified as one of the most important events in the reproduction of oviparous vertebrates. The genes for hatching enzymes, which are vital in the hatching process, are conserved among vertebrates. However, especially in teleost, it is difficult to trace their molecular evolution in detail due to the presence of other C6astacins, which are the subfamily to which the genes for hatching enzymes belong and are highly diverged. In particular, the hatching enzyme genes are diversified with frequent genome translocations due to retrocopy.

**Results:**

In this study, we took advantage of the rapid expansion of whole-genome data in recent years to examine the molecular evolutionary process of these genes in vertebrates. The phylogenetic analysis and the genomic synteny analysis revealed C6astacin genes other than the hatching enzyme genes, which was previously considered to be retained only in teleosts, was also retained in the genomes of basal ray-finned fishes, coelacanths, and cartilaginous fishes. These results suggest that the common ancestor of these genes can be traced back to at least the common ancestor of the Gnathostomata. Moreover, we also found that many of the C6astacin genes underwent multiple gene duplications during vertebrate evolution, and the results of gene expression analysis in frogs implied that genes derived from hatching enzyme genes underwent neo-functionalization.

**Conclusions:**

In this study, we describe in detail the molecular evolution of the C6astacin gene in vertebrates, which has not been summarized previously. The results revealed the presence of the previously unknown C6astacin gene in the basal-lineage of jawed vertebrates and large-scale gene duplication of hatching enzyme genes in amphibians. The comprehensive investigation reported in this study will be an important basis for studying the molecular evolution of the vertebrate C6astacin genes, hatching enzyme, and its paralogous genes and for identifying these genes without the need for gene expression and functional analysis.

**Supplementary Information:**

The online version contains supplementary material available at 10.1186/s12862-022-01966-2.

## Background

In fish and amphibians, the egg envelope, also referred to as the egg coat, egg membrane, or chorion, covers the embryos to protect them from environmental impacts such as physical stress and bacterial infection. At the end of embryogenesis, the embryos hatch out by tearing the egg envelope, with the assistance of hatching enzymes (HEs) secreted from the hatching gland cells [1, 2]. In reptiles and Aves, the egg envelope corresponds to the vitelline membrane which covers the egg yolk, while in mammals, it corresponds to the zona pellucida. All vertebrate HE genes investigated to date belong to the astacin-superfamily metalloproteases [3–6].

**Fig. 1:**
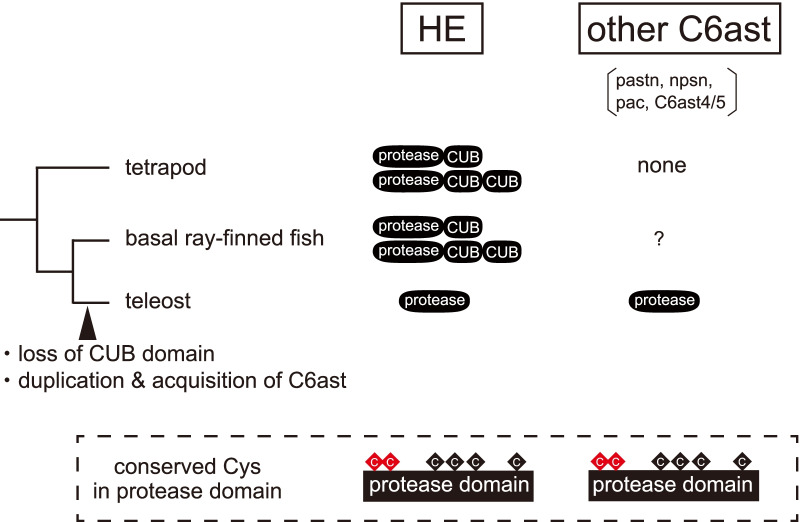
Molecular evolution of C6ast genes (HE, pastn, npsn, pac, and C6ast4/5) that have been considered to date. The molecular evolution of the C6ast genes is schematically shown. The C6ast genes have been identified to have two extra Cys (red diamonds in dotted box) in the protease domain compared to other astacin-family proteases having four conserved Cys residues (black diamonds in dotted box). The HE of non-teleostean fishes have CUB domain structures at the C-terminal side of astacin domain, like BMP1, while the HE and C6ast of teleosts have no domain structure at the C-terminal side of astacin domain. From the characteristics of these conserved Cys residues and domain structure, it has been previously thought that the C6ast gene arose by gene duplication of the HE gene in the common ancestor of teleosts

The astacin-superfamily genes are known to possess the consensus motifs HExxHxxGFxHExxRxDR and SxMHY at the active site for retaining a zinc, and four conserved Cys residues to maintain the higher-level conformation [7]. HE genes in vertebrates possess two extra conserved Cys residues, for a total of six Cys residues (Fig. 1). The gene family with this characteristic is called C6astacin (C6ast) [8]. During the evolution of teleosts, the C6ast genes have been duplicated, and they are classified into the subfamily, which contains the C6astacin4/5 (C6ast4/5), nephrosin (npsn), patristacin (pastn), pactacin (pac), and HEs [9].

The function of C6ast subfamily genes other than HE has already been investigated in several studies. The functions and expression patterns of these subfamily genes have diverged. It has been suggested that in zebrafish, npsn plays a role in biodefense, in neutrophils [10], while pac is expressed in the pancreas of seahorse and medaka and secreted into the intestine via the pancreatic duct, acting as a digestive enzyme [9]. It has been suggested that pastn (also called cimp1 in cichlid fish) may have contributed to the extension of the mandibular bones [11] and, further, to the parental internal brooding of eggs in Syngnathiformes and Cyprinodontiformes because they have divergent pastn genes [12–14]. C6ast4/5 genes are expressed in the jaw in adult medaka, although their function remains not well understood [8]. While the functions of other C6ast are diverse, the functions of teleost HEs are limited, since these genes are only expressed in hatching gland cells in pre-hatched embryos [1, 2].

Many astascin family metalloproteases retain the CUB (complement subcomponents C1r/C1s, embryonic sea urchin protein Uegf, BMP1) domain structure(s) at the C-terminal of the protease domain (Fig. 1) [15]. The HE genes of tetrapods also have this structure [5, 6]. We have recently found that the HE genes originally had CUB domain structures, which are not present in the common ancestor of teleosts [16]. Other C6ast has been found only in teleosts so far and has no domain structure at the C-terminus. It was therefore assumed that all C6ast subfamily genes arose by gene duplication of the HE genes, in the common ancestor of the teleosts. However, data for non-teleost fishes remains lacking, making the evolutionary origin of the C6ast gene family controversial.

The HE genes were also duplicated during the evolution of the teleosts, which then formed a multi-copy gene family [17]. Teleost HE genes were duplicated by retrocopy (retroduplication, i.e., the duplication of genes via mature mRNA) so the genomic location of the HE genes differ among lineages [4]. Due to the complexity of the genomic location of HE, and the high variation in copy number of C6ast subfamily genes, the evolutionary history of these genes is hard to disentangle. In order to accurately identify these genes in a genome sequence, it is necessary to carefully examine them using molecular phylogenetic and genomic synteny analysis.

The amount of high-quality vertebrate genome data continues to expand and is valuable for the investigation of the genes of the C6ast subfamily and their molecular evolution. In this study, to gain a deeper understanding of the molecular evolution of the C6ast subfamily genes in vertebrates, we conducted a comprehensive investigation of the genetic and genomic databases and their molecular evolution. We have found that: (1) the origin of the C6ast genes can be traced back to at least the common ancestor of Gnathostomata; (2) not only teleost HE, but also teleostean other C6ast and tetrapod HE underwent lineage-specific gene duplication several times; and (3) some of the duplicated HE genes in tetrapods acquired new functions (neo-functionalized genes), although the HE genes have been thought to act only on the degradation of the egg envelope. The detailed description of the evolution of these C6ast genes in this study will be an important basis for identifying the genes prior an in-depth gene expression and functional analysis.

## Results

### Identification of newly cloned C6ast genes in basal lineage fishes

**Fig. 2:**
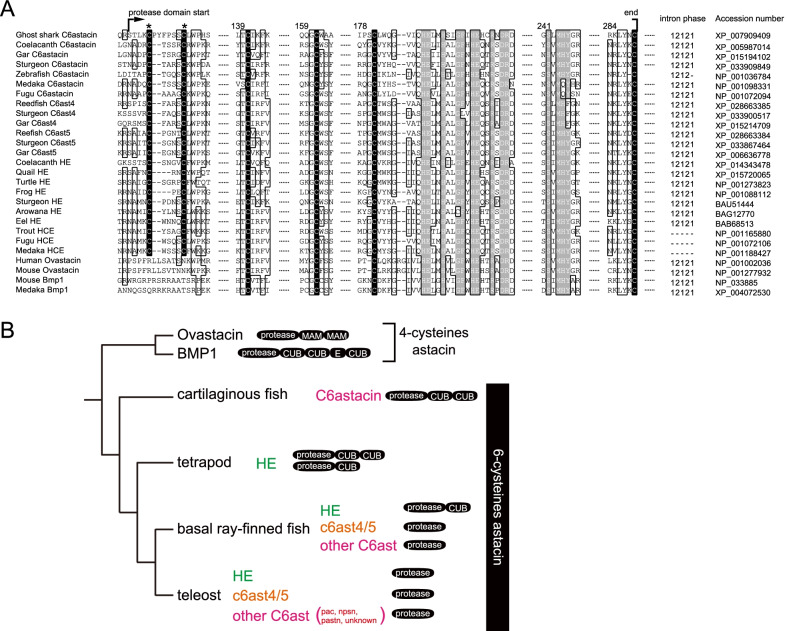
Comparisons of the primary structure of astacin-superfamily proteases. **A** Multiple alignments of amino acid sequences in representative astacin proteases. Only the sequences important for distinguishing the astacin proteases, the cysteine residues for disulfide bonds (highlighted with black), and the consensus sequence in the astacin-superfamily (highlighted with gray) are shown. The two Cys residues located at the NH_2_ terminal region of the protease domain (*) were highly conserved in all C6ast genes, including newly identified sequences. The exon–intron structure (intron phase) in the protease domain was also highly conserved, except for euteleostean HEs that are retrocopied genes. The position of the amino acids in coelacanth C6ast is shown above the alignment. **B** Schematic diagram of domain structure evolution of C6ast. The shape of the phylogenetic tree is illustrated based on previous studies and the tree shape in Additional file 1: Fig. S1. The black ellipses at the tips of the tree indicate the domain structure of each gene subfamilies (protease: protease domain, CUB: CUB domain, MAM: MAM domain, E: EGF-like domain)

We conducted a comprehensive search of the genome databases (Additional file 2: Table S1) and identified each relevant gene. We have found the C6ast genes other than HE in the genomes of the basal ray-finned fishes (gar, sturgeon, and reedfish), lobe-finned fish (coelacanth), and cartilaginous fishes, for the first time (Fig. 2). As shown in Fig. 2A, all of the C6ast genes in coelacanth, sturgeon, gar, reedfish and ghost shark retained six Cys residues, and the consensus sequences at the active site of the astacin-superfamily metalloprotease. The exon-intron structure of these genes was determined to be similar to that of teleost and tetrapod C6ast genes. Only teleost HE genes were lacking introns due to mRNA-mediated duplication, retrocopy. All of the newly identified genes have retained the characteristics of those in the primary sequences of the C6ast subfamily genes, which have already been identified.

We constructed a phylogenetic tree using representative astacin-superfamily, including BMP1, ovastacin and C6ast genes (Additional file 1: Fig. S1). Six cysteine conserved astacins were largely separated into C6ast4, C6ast5 (C6ast4/5), hatching enzyme (HE), nephrosin (npsn), pactacin (pac), and patristacin (pastn). HE formed a monophyletic clade with the other C6ast genes, indicating that the HE genes are members of the C6ast subfamily. The C6ast4/5 clade included the gar, sturgeon, and reedfish C6ast4/5, which are newly identified in this study. As shown in our previous study [10], pactacin genes constituted three subclades, and these subclades also comprised a single large clade, including pac and unknown genes, supported by high bootstrap values with unknown-C6ast in Otocephala and Protacanthopterygii. Since the bootstrap value at the node between unknown-C6ast and pac was low, this study did not reveal whether these unknown-C6ast are pac or not. Among the newly discovered genes, the C6ast genes of gar, sturgeon, and coelacanth fell into the outgroup of npsn, pac, and pastn, while the C6ast gene of cartilaginous fishes fell near C6ast4/5.

We have also identified new genes by analyzing their domain structure (Fig. 2B). The domain structures of HEs and other C6ast genes differ; the C-terminal of HE genes in bony fishes except teleosts (basal ray-finned fishes and tetrapods) was noted to possess one or two CUB domains, whereas none of other C6ast subfamily genes possess any C-terminal domains [3, 8]. Consistent with this feature, the novel gar and coelacanth C6ast genes did not have a CUB domain structure at the C-terminal region. Cartilaginous fishes, however, did have C6ast genes with a CUB domain at the C-terminal region.

In bony fishes, therefore, the characteristics of the novel C6ast genes—conservation of consensus sequences, the molecular phylogenetic tree, and the domain structure were consistent with the characteristics of previously identified C6ast genes [3, 9]. Cartilaginous fishes C6asts were found to retain the CUB domain structure, unlike the other C6ast found in other vertebrates. We have also analyzed the expression of newly identified genes using RT-PCR with RNA extracted from various adult tissues of gar, wherein we have found out that these genes are also expressed in several tissues (Additional file 1: Fig. S2). This expression pattern seems to be similar to that of the other C6ast genes expressed in various adult organs, rather than that of the HE genes, which are expressed only in embryos. These results suggested that the origin of C6ast subfamily genes dates back to at least the common ancestor of the jawed vertebrates. These results may also indicate that the common ancestor of the C6ast subfamily genes had one or more CUB domains.

### Analysis of genomic synteny

**Fig. 3:**
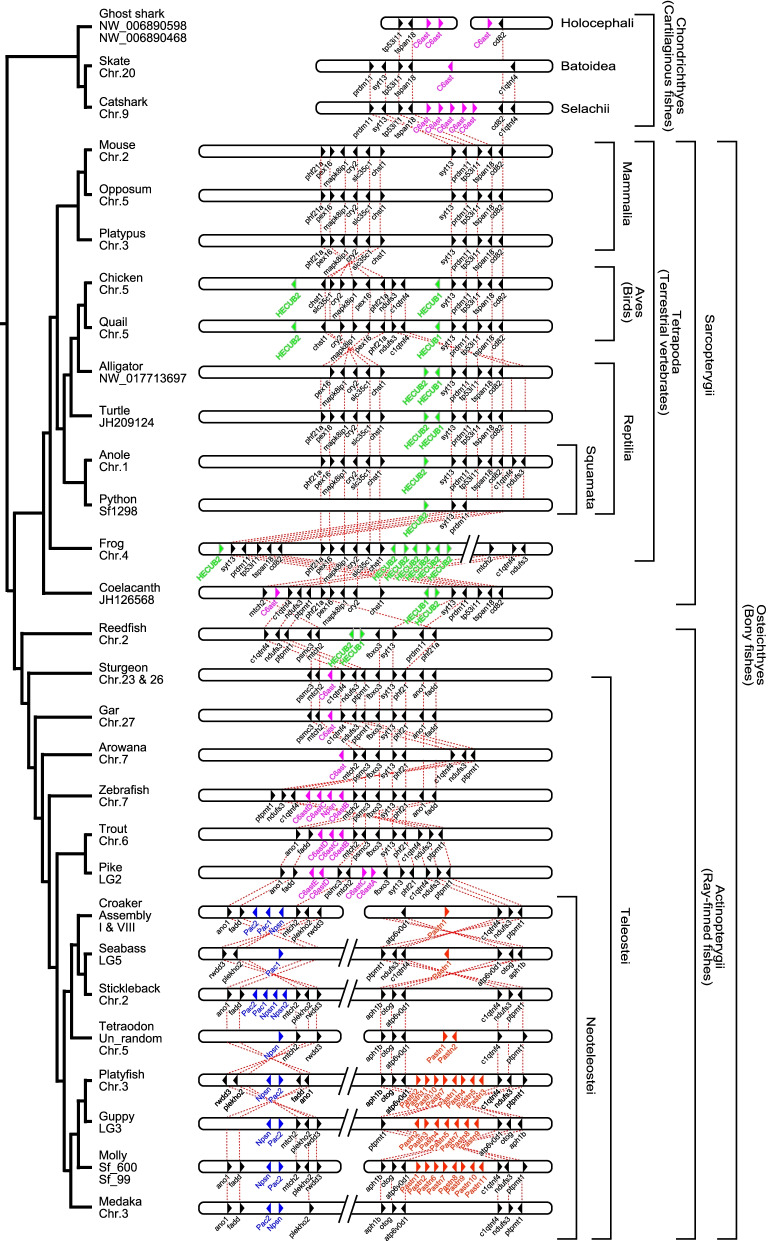
Syntenic evolution of HE and C6ast genes in vertebrates. The genomic synteny of the HE (green) and other C6ast (magenta, ancestral C6ast; red, patristacin; blue, nephrosin and pactacin) genes was strongly conserved during the evolution of the vertebrates. HECUB1 and HECUB2 indicate HE genes having a single or two CUB domain structures at the C-terminal side, respectively

We conducted a genomic synteny analysis of the C6ast-subfamily genes (Fig. 3). In Neotelestei, the genomic synteny of the pastn genes located in the vicinity of the *c1qtnf4*, *ndufs3*, and *ptpmt1* gene set, named the “Pastn-synteny-set,” was well conserved (red triangles in Fig. 3). Thus, pastn genes could be clearly distinguished from other C6ast-family genes by their genomic location, in addition to the phylogenetic analysis. Lin et al. and Small et al. have considered pastn to be related to internal egg brooding, because of the high copy number of patristacin in Syngnathiformes and ovoviviparous Cyprinodontiformes and the change of its expression levels in the brood pouch of pipefish observed between before and after pregnancy [13, 14]. However, our study found that oviparous Cyprinodontiformes also has a multi-copy gene family of pastn (Additional file 1: Fig. S1). Since these multiple pastn genes in Cyprinodontiformes occur in tandem, these genes seem to have been generated by tandem gene duplication.

The genomic synteny of npsn and pac (except for pac3) was highly conserved among the Neoteleostei (blue triangles in Fig. 3). These “Npsn&Pac-synteny-set” consisted of *mtch2*, *plekho2*, *fadd*, and *ano1* in tandem. “Npsn&Pac-synteny-set” and “Pat-synteny-set” were located on the same chromosome, at least in species in which the genome was assembled at the chromosomal level. Exceptionally, pac3 was located on another chromosome, but their genomic synteny was conserved among Euteleostei, which includes Neoteleostei pike and trout (Additional file 1: Fig. S3).

In non-Neoteleostean fishes (arowana, zebrafish, trout, and pike), some C6ast subfamily genes other than HEs were located close to each other (< 30 kbp distant in zebrafish) so they were placed between the Pastn- and Npsn&Pac-synteny-set (Fig. 3). The basal bony fishes (arowana, gar, sturgeon, and coelacanth) were noted to possess a single-copy C6ast gene at this locus. No C6ast genes, except for HE, were found here in reedfish, which belong to the Polypteriformes. Sturgeon exceptionally retained a single C6ast gene on each of the different chromosomes, and the surrounding synteny was also highly conserved (chr. 23 and 26). Since sturgeon underwent lineage-specific whole-genome duplication (WGD; 18), these gene pairs are thought to have arisen by WGD. However, we could not find any traces of C6ast gene pair derived from teleost specific WGD. These results suggested that the multiple copies of C6ast genes in teleostei were derived from a single C6ast gene. Cartilaginous fishes C6ast will be discussed later.

The C6ast4/5 genes were located tandemly between *anksf1* and *tra2b*, an arrangement which was conserved throughout the ray-finned fishes, including novel C6ast4/5 genes in basal ray-finned fishes (gar, sturgeon, and reedfish) (Additional file 1: Fig. S4). However, the C6ast4/5 genes were not found in the genomes of cartilaginous fishes, coelacanth, or tetrapods. Since C6ast4 and C6ast5 could be clearly distinguished in the phylogenetic analysis (supported by 99% bootstrap values), these results indicated that C6ast4 and C6ast5 were already differentiated in the common ancestor of the ray-finned fishes.

We have previously predicted the sequence of the HE genes in the Sarcopterygii from the genomic sequence [3]. The synteny analysis in this study revealed that HE genes were located in the vicinity of *syt13*, *prdm11*, *tp53l11*, *tspan18*, and *cd82* in Sarcopterygii, named the “HE-synteny-set” (Fig. 3). These results indicated that the genomic synteny of the HE genes was reasonably well conserved among sarcopterygian species (green triangle in Fig. 3), unlike the teleostean HE genes, which were translocated on the genome during evolution [4]. While most sarcopterygian HE genes were located close together on the same chromosome, their copy numbers varied from species to species, indicating that the variations of HE genes were caused by lineage-independent gene duplication.

The HE gene was not found in mammals, although the HE-synteny-set was noted to be well conserved. The HE gene was also not found in the platypus, which is an oviparous mammal. These results suggested that the HE gene was lost in the common ancestor of mammals. This loss of the HE gene is thought to be due to single gene loss, as it occurs despite the conservation of the surrounding synteny. On the other side, this syntenic relationship was consistent with that of the novel C6ast genes in cartilaginous fishes.

### Expression analysis of duplicated HE genes in frogs

**Fig. 4:**
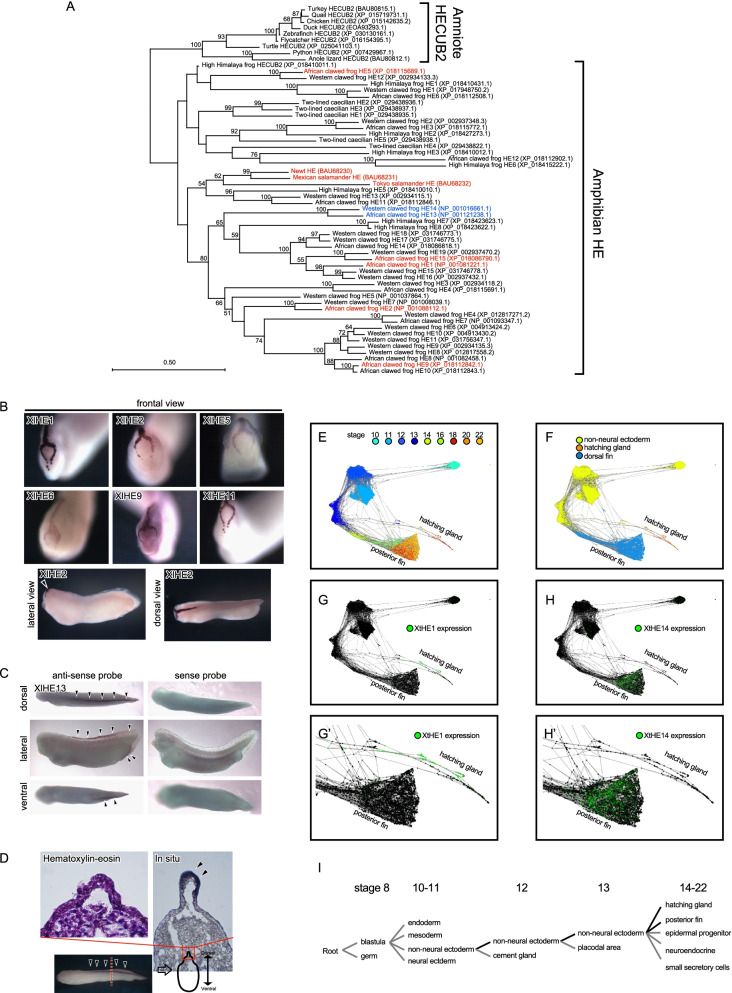
Gene expression pattern of the duplicated HEs in *Xenopus* frogs. **A** Phylogenetic tree of amphibian HEs constructed using the maximum likelihood method, with the amniote HEs as the outgroup (HECUB2 indicate HE genes having two CUB domain structures at the C-terminal side). Genes colored in red are genes whose expression in hatching gland cells has been confirmed in previous studies or this study. Genes that are colored blue (XlHE13 and XtHE14) were expressed at the caudal fin. The numbers at the nodes indicate the bootstrap values (> 50%). Whole mount in situ hybridization of HEs in hatching gland cells (**B**) and of XlHE13 genes in fins (**C**) and XlHE13 expression on the sections of caudal fins (**D**) in *X. laevis*. **E**–**H** Spatiotemporal reanalysis of XtHE1 and XtHE14 using t-distributed stochastic neighbor embedding with public data on the single-cell transcriptome of Western clawed frog (**E** developmental stages; **F** cell lineages; **G** and **G’**, expression of XtHE1; **H** and **H’**, expression of XtHE14) indicates different expression patterns in these genes. **I** Cell-fate tree of hatching gland and posterior fin estimated from the single-cell transcriptome. Each cell lineage was derived from the same origin, non-neural ectoderm

Teleostean HE genes form multi-copy gene families in several species. Sarcopterygii had multiple copies of genes in some species, and many HE genes were often detected, especially in frog (Western clawed frog: *Xenopus tropicalis*) (Fig. 3). In order to determine whether this large-scale duplication of the HE gene is specific to Western clawed frog, we searched the genomic data of three species of frogs, that is, Western clawed frog, African clawed frog *X. laevis*, and High Himalaya frog *Nanorana parkeri*, and one species of limbless amphibia (two-lined caecilian: *Rhinatrema bivittatum*), and performed a molecular phylogenetic analysis. We found that each species had 5 to 14 duplicated HE genes (Fig. 4A). Further genome synteny analysis revealed that all the HE genes were found in the HE-synteny-set in two-lined caecilian (Additional file 1: Fig. S5A), which is located in the phylogenetic outgroup of amphibians, while, in frogs, some HE genes were also located on different chromosomes (Additional file 1: Fig. S5B). The genus Xenopus is known to have undergone WGD during evolution, and African clawed frog is a heterotetraploid while Western clawed frog is a diploid, making it a model organism for examining the effects of WGD on molecular evolution [19]. However, Western clawed frog (19 genes), which did not experience WGD, retained a higher copy number of HE genes than African clawed frog (15 genes; Additional file 1: Fig. S6). These results seem to be inconsistent with WGD, but African clawed frog retained some traces of gene pairs acquired by doubling (Chr. 4L and 4S in Additional file 1: Fig. S5A), while Western clawed frog increased its copy number by tandem gene duplications.

We also investigated whether all these amphibian HE genes have six Cys residues in the protease domain. Although all vertebrate HE genes reported so far retain six Cys residues, African clawed frog HE1 exceptionally lacked two Cys residues at the N-terminal side of the protease domain [20]. Comparing all amphibian HE genes used in this study, most amphibian HE genes retained all six Cys residues, while it was revealed that some HE genes lost the Cys residues at the N-terminal side (Additional file 1: Fig. S6). These results indicate that some HE genes, including African clawed frog HE1, independently lost Cys residues. In African clawed frog, both HE2, which retains these Cys residues [21], and HE1, which has lost them [20], are known to function as hatching enzymes. In the future, comparing the protein interaction between these HEs and egg membrane may clarify the role of these two Cys resides that characterize the C6ast genes.

We then conducted an expression analysis of the diversified HE genes in frogs (Fig. 4B–H). In African clawed frog, XlHE1 (also known as UVS.2) [20] and XlHE2 [21] have already been identified as HE genes, and their spatial pattern of mRNA expression has already been reported. Our analysis also detected the expression of both XlHE1 and XlHE2 in hatching gland cells localized as inverted-Y shapes in the dorsal head region before the hatching stage (NF stage 36; Fig. 4B). Although we failed to detect the expression of some hatching enzyme-like genes, similar patterns of expression were detected in XlHE5, 6, 9, and 11 (Fig. 4B). In contrast, the expression of XlHE13 was detected in the posterior fin after the hatching stage (NF stage 40; Fig. 4C). We also confirmed the expression localization in this posterior fin from the single-cell transcriptome (scRNAseq) data of Western clawed frog (Fig. 4E–H)—from phylogenetic analysis and synteny analysis, XlHE13 is homologous to XtHE14 (*X. tropicalis* HE14). After hatching, the embryos are not covered by the egg membrane, which is a substrate for the HEs. Since XlHE13 was expressed in the epidermal tissue layer, rather than in the head where the hatching gland cells are localized, it seems that XlHE13 has a different role from that of the HEs. These results indicate that the duplicate gene derived from the HE genes has acquired new functions. Cell lineage analysis using scRNAseq data from Western clawed frog showed that hatching gland cells and posterior fins follow a similar cell lineage, derived from non-neural ectoderm (Fig. 4I). Gene duplication may have caused subtle changes in the transcriptional regulation, leading to the acquisition of new functions by the frog’s HE gene.

## Discussion

**Fig. 5:**
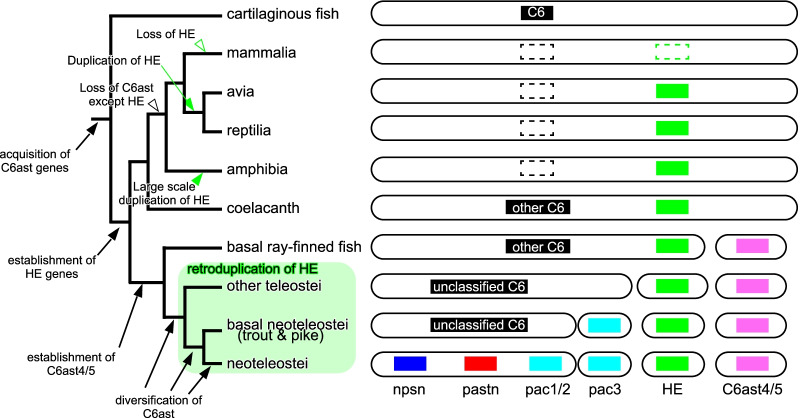
Schematic representation of the molecular evolution of C6ast genes. The outline of the species lineage is shown in association with the presence or lacking (solid and dotted squares, respectively) of C6ast genes. The rounded rectangles schematically represent chromosomes, and genes located on the same chromosome are drawn on the same rounded rectangles. The arrows and arrowheads indicate the timing of loss acquisition, establishment or duplication of each genes

We provide a detailed description of the molecular evolution of six cysteine conserved astacins in vertebrates (summarized in Fig. 5). We believe that this study will provide a molecular basis for the study of six cysteine conserved astacin genes.

### The evolutionary origin of the HE and C6ast

This study prompts a reconsideration of the evolutionary origins of six cysteine conserved astacins. We were able to find the C6ast gene in the shark genome. However, since the expression of these genes has not yet been analyzed, the function of these genes is unclear. We also found a C6ast-like fragment sequence (XP_020386440) downstream of the *cd82* gene (XP_020386441) in the genome of the whale shark, which is well known for its viviparity. Although the HE genes have become pseudogenes and are fragmented in many ovoviviparous fishes, it is not possible to determine whether this fragmentation indicates pseudogenization, due to the insufficient quality of the published whale shark genome. Thus, in the future, a more detailed genome sequence is needed, which will be available soon; [22], as well as an expression analysis using embryos.

However, we could not find the astacin gene with six Cys residues in the genome of the jawless fish, even the “HE-synteny-set”. The HE and C6ast genes were located on the same chromosome and were in close proximity to each other, especially in the basal lineage. These results imply that this organization probably arouse by tandem gene duplication rather than being ohnologs produced by WGD. However, these results do not indicate that HE and C6ast both occurred in the common ancestor of jawed vertebrates, because there is still the possibility that the jawless fish lost both genes independently.

### Effect of whole genome duplication on the molecular evolution of the C6ast gene

We found a C6ast gene pair derived from WGD in sturgeon and African clawed frog. However, we could not find any trace of the teleost-specific WGD on the evolution of the C6ast gene. It is known that many gene pairs derived from WGD rapidly revert to single genes due to their redundancy and reconstruction of chromosome [23]. The long evolutionary time (300MY) that has passed since teleost specific WGD occurred may have led to genomic reorganization at the chromosomal level. Therefore, the synteny of the other C6ast gene that duplicated by teleost specific WGD may not be followed.

### Gene loss of HE genes in mammalian lineage

We have determined that mammals lack the HE gene, which is homologous to the hatching enzymes in fish and amphibians. In the eutheria, the zona pellucida corresponds to the egg envelope of fish and amphibians [24], and local degradation of the zona pellucida in mammals during implantation corresponds to hatching. It is believed that trypsin-like protease, not astacin-family protease, is responsible for this local degradation [25, 26]. Therefore, the lack of genes homologous to HE in fish and frogs in mammals may be attributable to the shift of the zona pellucida-degrading enzyme to trypsin-like, as the oviparous to viviparous change occurred. However, our results show that platypus lost the HE gene, and they are likely to have lost the HE gene before the shift into viviparity. To the best of our knowledge, molecular level examinations into the hatching process of platypus have not yet been conducted. Our results are expected to be useful in the investigation of the co-evolution of HEs and egg membrane proteins in mammalian lineages.

### Lineage-specific duplication and neo-functionalization of C6ast and HE genes

This study revealed that HE and C6ast genes have undergone several lineage-specific gene duplications and neo-functionalization during vertebrate evolution. In particular, we have determined that one of the HE genes in frogs (XlHE13 and XtHE14) had neo-functionalized (Fig. 4). BMP1 belongs to the astacin-superfamily and is known to be vital in the formation of the activity gradients of other BMPs belonging to the TGF-β superfamily. BMP1 digests chordin and noggin, which are BMP inhibitors. It has been suggested that chicken ASTL (astacin-like), corresponding to HECUB2 in this paper (Fig. 3 and Additional file 1: Fig. S1), arose by duplication of the hatching enzyme, HECUB1 in this paper, and is as important for the formation of BMP activity gradients as BMP1 [27]. The frog XlHE13 and XtHE14 may have similar functionality. BMP2, BMP4, and noggin in frogs are similarly expressed in the posterior fin [28, 29]. In order to examine whether XlHE13 is important for the formation of frog fin buds, research into the function of the gene, using gene knockout, will be needed. It appears that the tetrapod HE gene and the teleost C6ast diversified their functions by neo-functionalization due to gene duplication in their lineages.

Teleostean HE expression and function have been determined to be limited, being expressed only in hatching gland cells before hatching and functioning to degrade the main component of the egg envelope, the zona pellucida protein (ZP protein) [24, 30]. Although teleostean HE is specialized in degrading ZP protein, the recognition and cleavage specificity of ZP protein are changed [31]. Teleostean HE, therefore, had diversified substrate specificity, while maintaining its specialized role in egg envelope degradation, in order to adapt to the variety of the teleost egg envelope. To elucidate the mechanism underlying the ease with which HE and other C6ast change substrate specificity, it will be important to identify the residues important for three-dimensional structural interactions between astacin-family proteases and the substrate and to investigate their molecular evolution.

## Conclusions

In this study, we used a large amount of genomic data currently available and a variety of approaches to investigate the molecular evolution of hatching enzyme genes in teleost fish. Our study findings show that genes had been duplicated and acquired new function many times over the course of evolution and the common ancestor of these genes has been traced back to at least the common ancestor of the Gnathostomata (the jawed vertebrates). These data provide a sound basis for future research in this area.

## Methods

### Animals

Adult African clawed frog (*X. laevis*) were obtained from a commercial supplier (Kazuo Ouchi biomaterial supplier, Saitama, Japan) and kept in our laboratory. The artificial insemination of African clawed frog was conducted using the same method as described in our previous study [3]. The eggs were kept and raised in freshwater until the frogs reached specific developmental stages, determined via conventional criteria [32], at which time the total RNA was collected or the embryos or larvae were fixed.

### In silico cloning and comparison of genomic synteny

The methods of obtaining sequences, in silico cloning, and comparison of genomic synteny were as previously described [3], with slight modifications, as described below. For in silico cloning, a BLAST search was performed, using the sequences of closely related species as a query, on the genomic data obtained from the NCBI genome database [33] (Additional file 2: Table S1). The exon sequences were cut out from the sequence around the hit region predicted using GeneWise 2 [34], or by visually observing multiple alignments according to the GT-AG rule [35]. The genomic synteny analysis was conducted using Genomicus ver. 98.01 [36] and the Genome Data Viewer [37], except for unregistered species. In unregistered species, synteny analysis was conducted using homology search against downloaded genome sequences, with the homolog sequences from closely related species as a query. All genomic databases used in this study are listed in Additional file 2: Table S1.

### Multiple alignments and construction of phylogenetic trees

Multiple alignments of the amino acid sequences of the astacin proteases were constructed using ClustalX [38] or MAFFT [39]. The phylogenetic trees were drawn using the maximum likelihood method with RAxML [40] or the neighbor joining method using MEGA X [41], using the aligned sequences of the mature enzymatic region. The bootstrap values were calculated using 1000 replicates.

### In situ hybridization and hematoxylin and eosin staining

Whole-mount in situ hybridization (WISH) was performed according to a previously published method [42]. Fixed samples of African clawed frog were breached and refixed before the WISH procedure. The stained samples were embedded in optimal cutting temperature compound and were then sectioned using a cryostat. For histological observation, fixed samples were dehydrated in ethanol series and embedded into paraffin via xylene. The embedded samples were sectioned, deparaffinized, and stained with hematoxylin and eosin, in accordance with the standard method.

### Reanalysis of single-cell RNA sequencing

The previously sequenced single-cell RNA-seq data [43] were obtained from Xenopus Jamboree (https://kleintools.hms.harvard.edu/tools/currentDatasetsList_xenopus_v2.html). For reanalysis, only the normalized data pertaining to the cell lineage of the hatching gland cells and the caudal fin distinguished by the combination of t-distributed stochastic neighbor embedding and the expression of selected marker proteins were used. Cell clustering was performed using t-distributed stochastic neighbor embedding, as in the previous study [43], and the gene expression level was visualized. The cell-fate tree was obtained from the Xenopus Jamboree and simplified.

## Supplementary Information

**Additional file 1: Figure S1.** Phylogenetic tree of the astacin proteases. The phylogenetic relations were calculated using neighbor joining method after producing multiple alignments of the amino acid sequences in the mature portion of the enzyme by using MAFFT program. The numbers at the nodes indicate the bootstrap values (> 50%). HECUB1 and HECUB2 indicate HE genes having a single or two CUB domain structures at the C-terminal side, respectively. This tree is summarized in Fig. 2B. **Figure S2.** RT-PCR analysis of the C6ast gene in adult organs from gar. RNA was extracted from each organ in adult gar, and RT-PCR was performed (26 amplification cycles). A clear amplified band in the liver and weak amplification bands in the heart, stomach, intestines, kidneys, and spleen were identified (upper panel). The lower panel indicates the amplified product of β-actin for positive control. **Figure S3.** Genomic synteny of the pactacin3 genes. The schematic drawing of genomic synteny in pactacin3 (red triangles) is shown in the same format in Fig. 3. **Figure S4.** Genomic synteny of the C6ast4/5 genes. The schematic drawing of genomic synteny in C6ast4/5 (red triangles) is shown in the same format in Figure S3. **Figure S5.** Genomic synteny of HE genes in Amphibia. As described in Fig. 3, the synteny of the Western clawed frog HE genes are conserved between other vertebrates. (A) The synteny of the HE genes (green triangles) which is consistent with that of other tetrapods and (B) that of the amphibian-specific type. **Figure S6.** The multiple aligned amino acid sequences in the protease domain of the amphibian HE genes. The consensus sequence was highlighted as in Fig. 1, and only the two Cys residues on the N-terminal side were highlighted in red.

**Additional file 2: Table S1.** Species used for genomic comparison.

## Data Availability

The nucleotide sequence data reported in this study will appear in the DDBJ/EMBL/GenBank nucleotide sequence databases under accession numbers LC632423- LC632429, LC637434- LC637435.
