# Supplementary material for: Molecular evolution of hatching enzymes and their paralogous genes in vertebrates
Source: BMC Ecol Evol. 2022 Feb 2;22:9. doi: 10.1186/s12862-022-01966-2 (PMC8812170; doi:10.1186/s12862-022-01966-2)
Supplement: Supplementary file 1 — Additional file 1: Figure S1. Phylogenetic tree of the astacin proteases. The phylogenetic relations were calculated using neighbor joining method after producing multiple alignments of the amino acid sequences in the mature portion of the enzyme by using MAFFT program. The numbers at the nodes indicate the bootstrap values (> 50%). HECUB1 and HECUB2 indicate HE genes having a single or two CUB domain structures at the C-terminal side, respectively. This tree is summarized in Fig. 2B. Figure S2. RT-PCR analysis of the C6ast gene in adult organs from gar. RNA was extracted from each organ in adult gar, and RT-PCR was performed (26 amplification cycles). A clear amplified band in the liver and weak amplification bands in the heart, stomach, intestines, kidneys, and spleen were identified (upper panel). The lower panel indicates the amplified product of β-actin for positive control. Figure S3. Genomic synteny of the pactacin3 genes. The schematic drawing of genomic synteny in pactacin3 (red triangles) is shown in the same format in Fig. 3. Figure S4. Genomic synteny of the C6ast4/5 genes. The schematic drawing of genomic synteny in C6ast4/5 (red triangles) is shown in the same format in Figure S3. Figure S5. Genomic synteny of HE genes in Amphibia. As described in Fig. 3, the synteny of the Western clawed frog HE genes are conserved between other vertebrates. (A) The synteny of the HE genes (green triangles) which is consistent with that of other tetrapods and (B) that of the amphibian-specific type. Figure S6. The multiple aligned amino acid sequences in the protease domain of the amphibian HE genes. The consensus sequence was highlighted as in Fig. 1, and only the two Cys residues on the N-terminal side were highlighted in red. [file 12862_2022_1966_MOESM1_ESM.pdf]

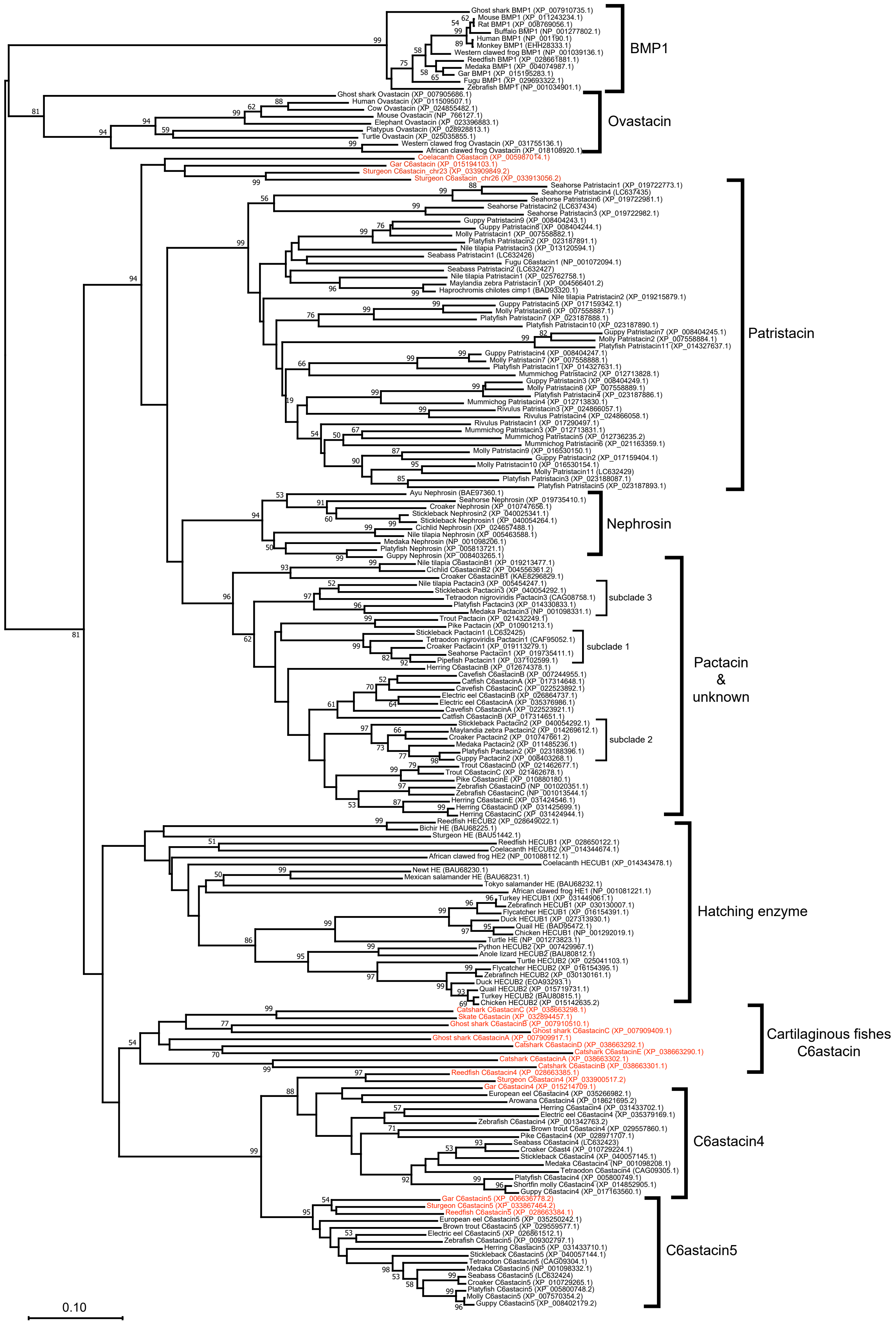
Ghost shark BMP1 (XP_007910735.1)
Mouse BMP1 (XP_011243234.1)
Rat BMP1 (XP_008769056.1)
Buffalo BMP1 (NP_001277802.1)
Human BMP1 (NP_001190.1)
Monkey BMP1 (EHH28333.1)
Western clawed frog BMP1 (NP_001039136.1)
Reedfish BMP1 (XP_028661881.1)
Medaka BMP1 (XP_004074987.1)
Gar BMP1 (XP_015195283.1)
Fugu BMP1 (XP_029693322.1)
Zebrafish BMP1 (NP_001034901.1)
BMP1
Ghost shark Ovastacin (XP_007905686.1)
Human Ovastacin (XP_011509507.1)
Cow Ovastacin (XP_024855482.1)
Mouse Ovastacin (NP_766127.1)
Elephant Ovastacin (XP_023396883.1)
Platypus Ovastacin (XP_028928813.1)
Turtle Ovastacin (XP_025035855.1)
Western clawed frog Ovastacin (XP_031755136.1)
African clawed frog Ovastacin (XP_018108920.1)
Ovastacin
Coelacanth C6astacin (XP_005987014.1)
Gar C6astacin (XP_015194103.1)
Sturgeon C6astacin_chr23 (XP_033909849.2)
Sturgeon C6astacin_chr26 (XP_033913056.2)
Seahorse Patristacin1 (XP_019722773.1)
Seahorse Patristacin4 (LC637435)
Seahorse Patristacin6 (XP_019722981.1)
Seahorse Patristacin2 (LC637434)
Seahorse Patristacin3 (XP_019722982.1)
Guppy Patristacin9 (XP_008404243.1)
Guppy Patristacin8 (XP_008404244.1)
Molly Patristacin1 (XP_007558882.1)
Platyfish Patristacin2 (XP_023187891.1)
Nile tilapia Patristacin3 (XP_013120594.1)
Seabass Patristacin1 (LC632426)
Fugu C6astacin1 (NP_001072094.1)
Seabass Patristacin2 (LC632427)
Nile tilapia Patristacin1 (XP_025762758.1)
Maylandia zebra Patristacin1 (XP_004566401.2)
Haprochromis chilotes cimp1 (BAD93320.1)
Nile tilapia Patristacin2 (XP_019215879.1)
Guppy Patristacin5 (XP_017159342.1)
Molly Patristacin6 (XP_007558887.1)
Platyfish Patristacin7 (XP_023187888.1)
Platyfish Patristacin10 (XP_023187890.1)
Guppy Patristacin7 (XP_008404245.1)
Molly Patristacin2 (XP_007558884.1)
Platyfish Patristacin11 (XP_014327637.1)
Guppy Patristacin4 (XP_008404247.1)
Molly Patristacin7 (XP_007558888.1)
Platyfish Patristacin1 (XP_014327631.1)
Mummichog Patristacin2 (XP_012713828.1)
Guppy Patristacin3 (XP_008404249.1)
Molly Patristacin8 (XP_007558889.1)
Platyfish Patristacin4 (XP_023187886.1)
Mummichog Patristacin4 (XP_012713830.1)
Rivulus Patristacin3 (XP_024866057.1)
Rivulus Patristacin4 (XP_024866058.1)
Rivulus Patristacin1 (XP_017290497.1)
Mummichog Patristacin3 (XP_012713831.1)
Mummichog Patristacin5 (XP_012736235.2)
Mummichog Patristacin6 (XP_021163359.1)
Molly Patristacin9 (XP_016530150.1)
Guppy Patristacin2 (XP_017159404.1)
Molly Patristacin10 (XP_016530154.1)
Molly Patristacin11 (LC632429)
Platyfish Patristacin3 (XP_023188087.1)
Platyfish Patristacin5 (XP_023187893.1)
Patristacin
Ayu Nephrosin (BAE97360.1)
Seahorse Nephrosin (XP_019735410.1)
Croaker Nephrosin (XP_010747656.1)
Stickleback Nephrosin2 (XP_040025341.1)
Stickleback Nephrosin1 (XP_040054264.1)
Cichlid Nephrosin (XP_024657488.1)
Nile tilapia Nephrosin (XP_005463588.1)
Medaka Nephrosin (NP_001098206.1)
Platyfish Nephrosin (XP_005813721.1)
Guppy Nephrosin (XP_008403265.1)
Nephrosin
Nile tilapia C6astacinB1 (XP_019213477.1)
Cichlid C6astacinB2 (XP_004556361.2)
Croaker C6astacinB1 (KAE8296829.1)
Nile tilapia Pactacin3 (XP_005454247.1)
Stickleback Pactacin3 (XP_040054292.1)
Tetraodon nigroviridis Pactacin3 (CAG08758.1)
Platyfish Pactacin3 (XP_014330833.1)
Medaka Pactacin3 (NP_001098331.1)
subclade 3
Trout Pactacin (XP_021432249.1)
Pike Pactacin (XP_010901213.1)
Stickleback Pactacin1 (LC632425)
Tetraodon nigroviridis Pactacin1 (CAF95052.1)
Croaker Pactacin1 (XP_019113279.1)
Seahorse Pactacin1 (XP_019735411.1)
Pipefish Pactacin1 (XP_037102599.1)
subclade 1
Herring C6astacinB (XP_012674378.1)
Cavefish C6astacinB (XP_007244955.1)
Catfish C6astacinA (XP_017314648.1)
Cavefish C6astacinC (XP_022523892.1)
Electric eel C6astacinB (XP_026864737.1)
Electric eel C6astacinA (XP_035376986.1)
Cavefish C6astacinA (XP_022523921.1)
Catfish C6astacinB (XP_017314651.1)
Stickleback Pactacin2 (XP_040054292.1)
Maylandia zebra Pactacin2 (XP_014269612.1)
Croaker Pactacin2 (XP_010747661.2)
Medaka Pactacin2 (XP_011485236.1)
Platyfish Pactacin2 (XP_023188396.1)
Guppy Pactacin2 (XP_008403268.1)
subclade 2
Trout C6astacinD (XP_021462677.1)
Trout C6astacinC (XP_021462678.1)
Pike C6astacinE (XP_010880180.1)
Zebrafish C6astacinD (NP_001020351.1)
Zebrafish C6astacinC (NP_001013544.1)
Herring C6astacinE (XP_031424546.1)
Herring C6astacinD (XP_031425699.1)
Herring C6astacinC (XP_031424944.1)
Pactacin
&
unknown
Reedfish HECUB2 (XP_028649022.1)
Bichir HE (BAU68225.1)
Sturgeon HE (BAU51442.1)
Reedfish HECUB1 (XP_028650122.1)
Coelacanth HECUB2 (XP_014344674.1)
African clawed frog HE2 (NP_001088112.1)
Coelacanth HECUB1 (XP_014343478.1)
Newt HE (BAU68230.1)
Mexican salamander HE (BAU68231.1)
Tokyo salamander HE (BAU68232.1)
African clawed frog HE1 (NP_001081221.1)
Turkey HECUB1 (XP_031449061.1)
Zebrafinch HECUB1 (XP_030130007.1)
Flycatcher HECUB1 (XP_016154391.1)
Duck HECUB1 (XP_027313930.1)
Quail HE (BAD95472.1)
Chicken HECUB1 (NP_001292019.1)
Turtle HE (NP_001273823.1)
Python HECUB2 (XP_007429967.1)
Anole lizard HECUB2 (BAU80812.1)
Turtle HECUB2 (XP_025041103.1)
Flycatcher HECUB2 (XP_016154395.1)
Zebrafinch HECUB2 (XP_030130161.1)
Duck HECUB2 (EOA93293.1)
Quail HECUB2 (XP_015719731.1)
Turkey HECUB2 (BAU80815.1)
Chicken HECUB2 (XP_015142635.2)
Hatching enzyme
Catshark C6astacinC (XP_038663298.1)
Skate C6astacin (XP_032894457.1)
Ghost shark C6astacinB (XP_007910510.1)
Ghost shark C6astacinC (XP_007909409.1)
Ghost shark C6astacinA (XP_007909917.1)
Catshark C6astacinD (XP_038663292.1)
Catshark C6astacinE (XP_038663290.1)
Catshark C6astacinA (XP_038663302.1)
Catshark C6astacinB (XP_038663301.1)
Cartilaginous fishes
C6astacin
Reedfish C6astacin4 (XP_028663385.1)
Sturgeon C6astacin4 (XP_033900517.2)
Gar C6astacin4 (XP_015214709.1)
European eel C6astacin4 (XP_035266982.1)
Arowana C6astacin4 (XP_018621695.2)
Herring C6astacin4 (XP_031433702.1)
Electric eel C6astacin4 (XP_035379169.1)
Zebrafish C6astacin4 (XP_001342763.2)
Brown trout C6astacin4 (XP_029557860.1)
Pike C6astacin4 (XP_028971707.1)
Seabass C6astacin4 (LC632423)
Croaker C6ast4 (XP_010729224.1)
Stickleback C6astacin4 (XP_040057145.1)
Medaka C6astacin4 (NP_001098208.1)
Tetraodon C6astacin4 (CAG09305.1)
Platyfish C6astacin4 (XP_005800749.1)
Shortfin molly C6astacin4 (XP_014852905.1)
Guppy C6astacin4 (XP_017163560.1)
C6astacin4
Gar C6astacin5 (XP_006636778.2)
Sturgeon C6astacin5 (XP_033867464.2)
Reedfish C6astacin5 (XP_028663384.1)
European eel C6astacin5 (XP_035250242.1)
Brown trout C6astacin5 (XP_029559577.1)
Electric eel C6astacin5 (XP_026861512.1)
Zebrafish C6astacin5 (XP_009302797.1)
Herring C6astacin5 (XP_031433710.1)
Stickleback C6astacin5 (XP_040057144.1)
Tetraodon C6astacin5 (CAG09304.1)
Medaka C6astacin5 (NP_001098332.1)
Seabass C6astacin5 (LC632424)
Croaker C6astacin5 (XP_010729265.1)
Platyfish C6astacin5 (XP_005800748.2)
Molly C6astacin5 (XP_007570354.2)
Guppy C6astacin5 (XP_008402179.2)
C6astacin5
0.10

Fig. S1

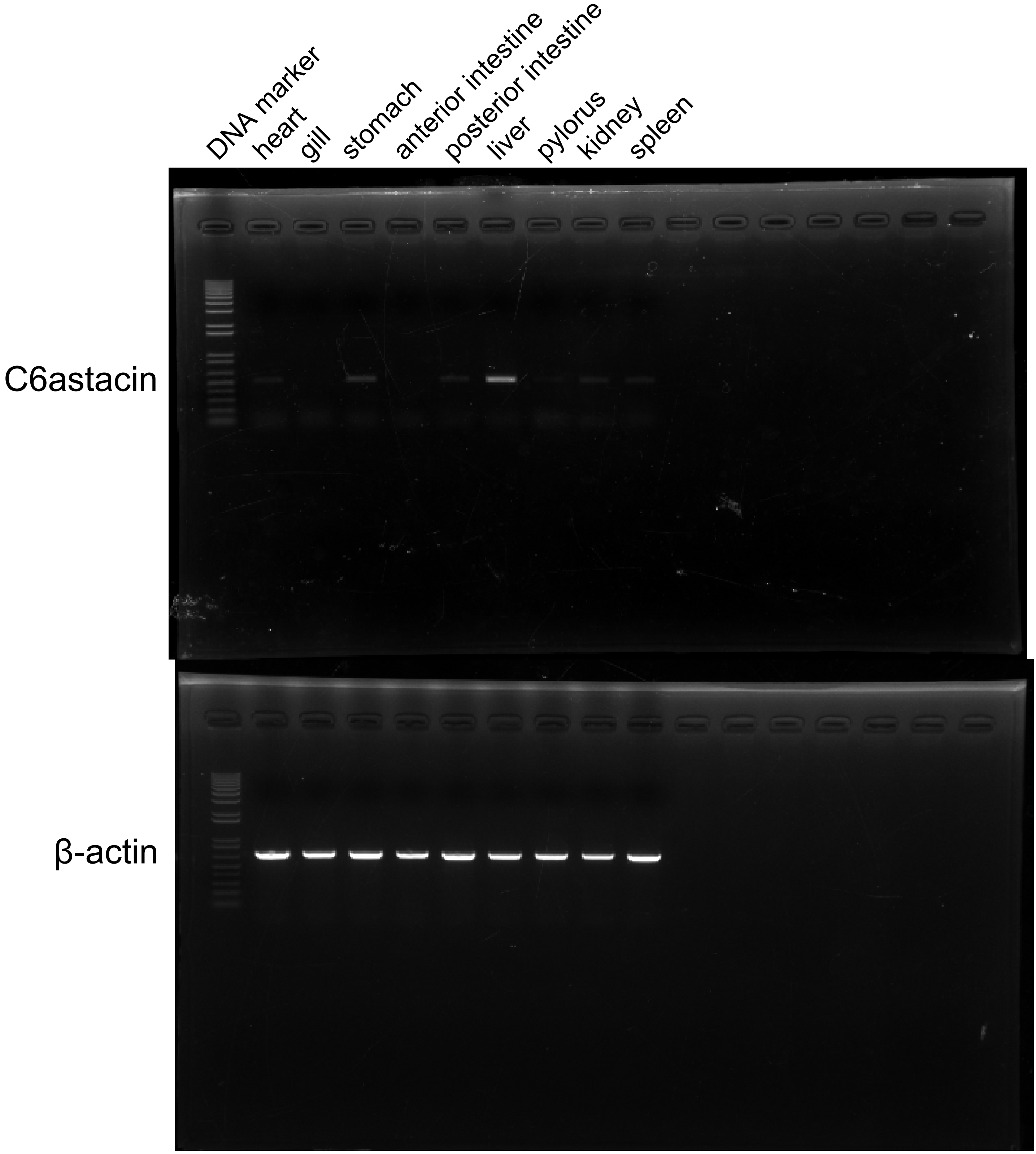

DNA marker
heart
gill
stomach
anterior intestine
posterior intestine
liver
pylorus
kidney
spleen
C6astacin
β-actin

Fig. S2

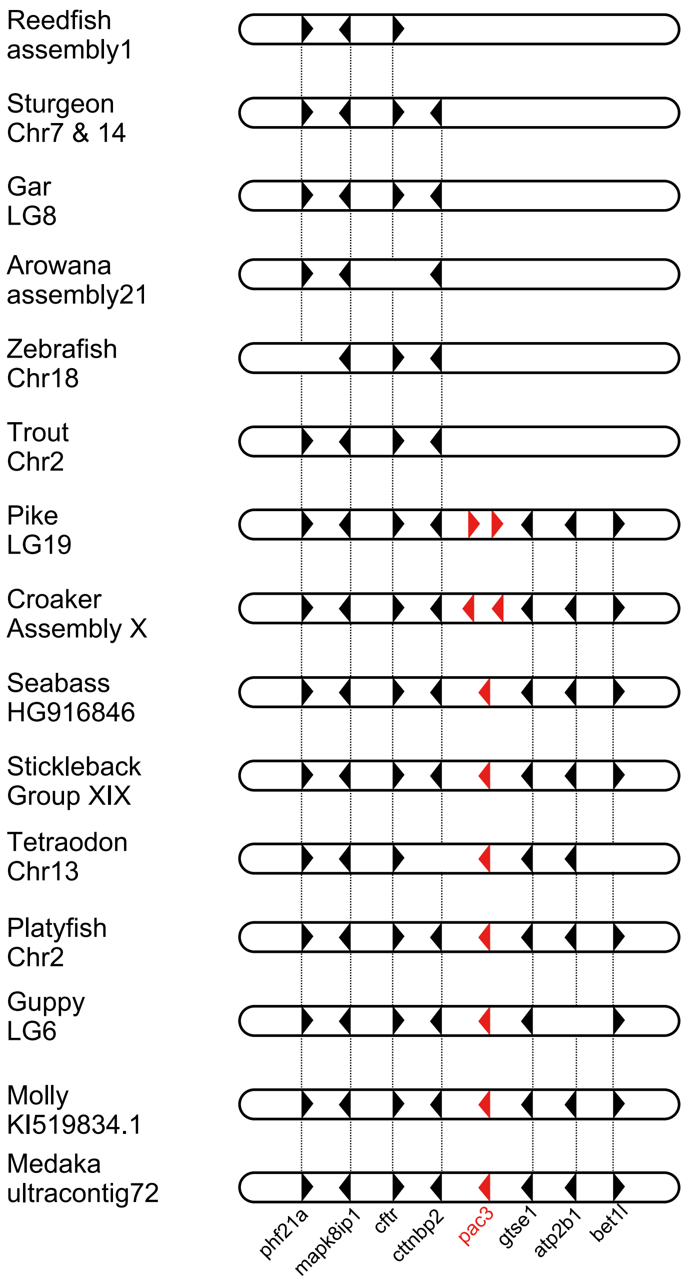
Reedfish
assembly1
Sturgeon
Chr7 & 14
Gar
LG8
Arowana
assembly21
Zebrafish
Chr18
Trout
Chr2
Pike
LG19
Croaker
Assembly X
Seabass
HG916846
Stickleback
Group XIX
Tetraodon
Chr13
Platyfish
Chr2
Guppy
LG6
Molly
KI519834.1
Medaka
ultracontig72
phf21a
mapk8ip1
cftr
cttnbp2
pac3
gtse1
atp2b1
bet1l

Fig. S3

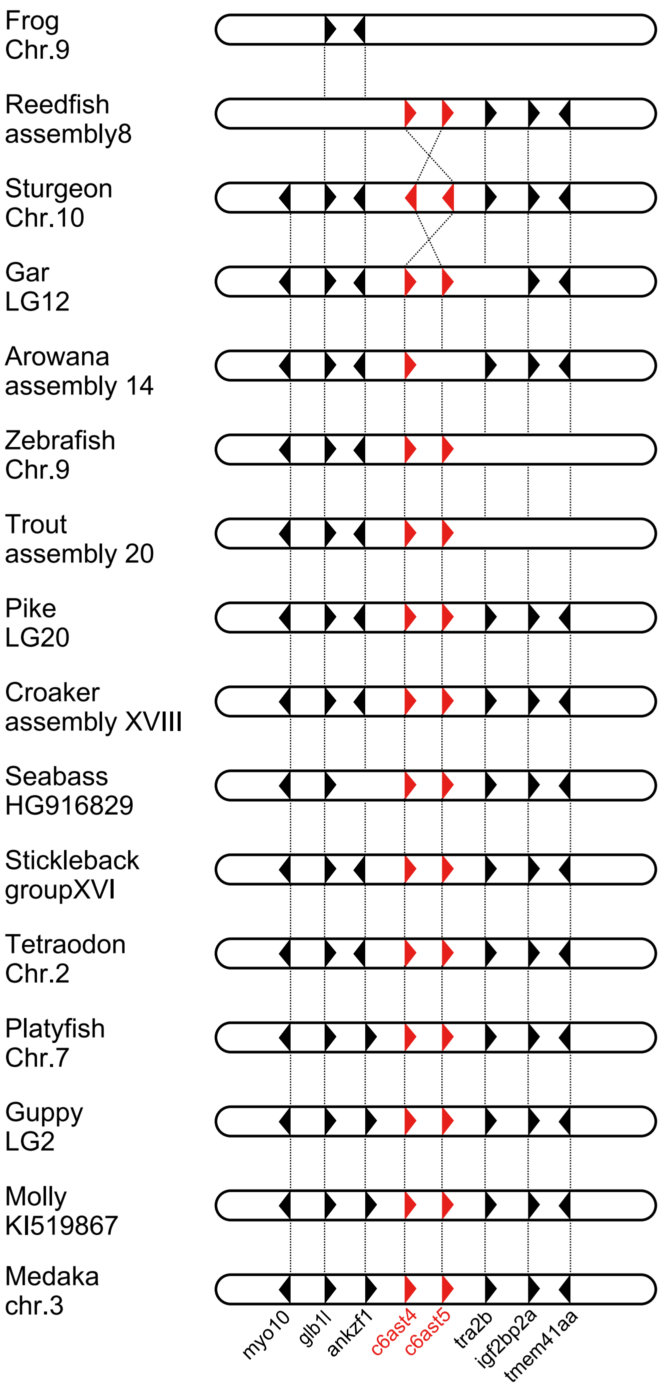
Frog
Chr.9
Reedfish
assembly8
Sturgeon
Chr.10
Gar
LG12
Arowana
assembly 14
Zebrafish
Chr.9
Trout
assembly 20
Pike
LG20
Croaker
assembly XVIII
Seabass
HG916829
Stickleback
groupXVI
Tetraodon
Chr.2
Platyfish
Chr.7
Guppy
LG2
Molly
KI519867
Medaka
chr.3
myo10
glb1l
ankzf1
c6ast4
c6ast5
tra2b
igf2bp2a
tmem41aa

Fig. S4

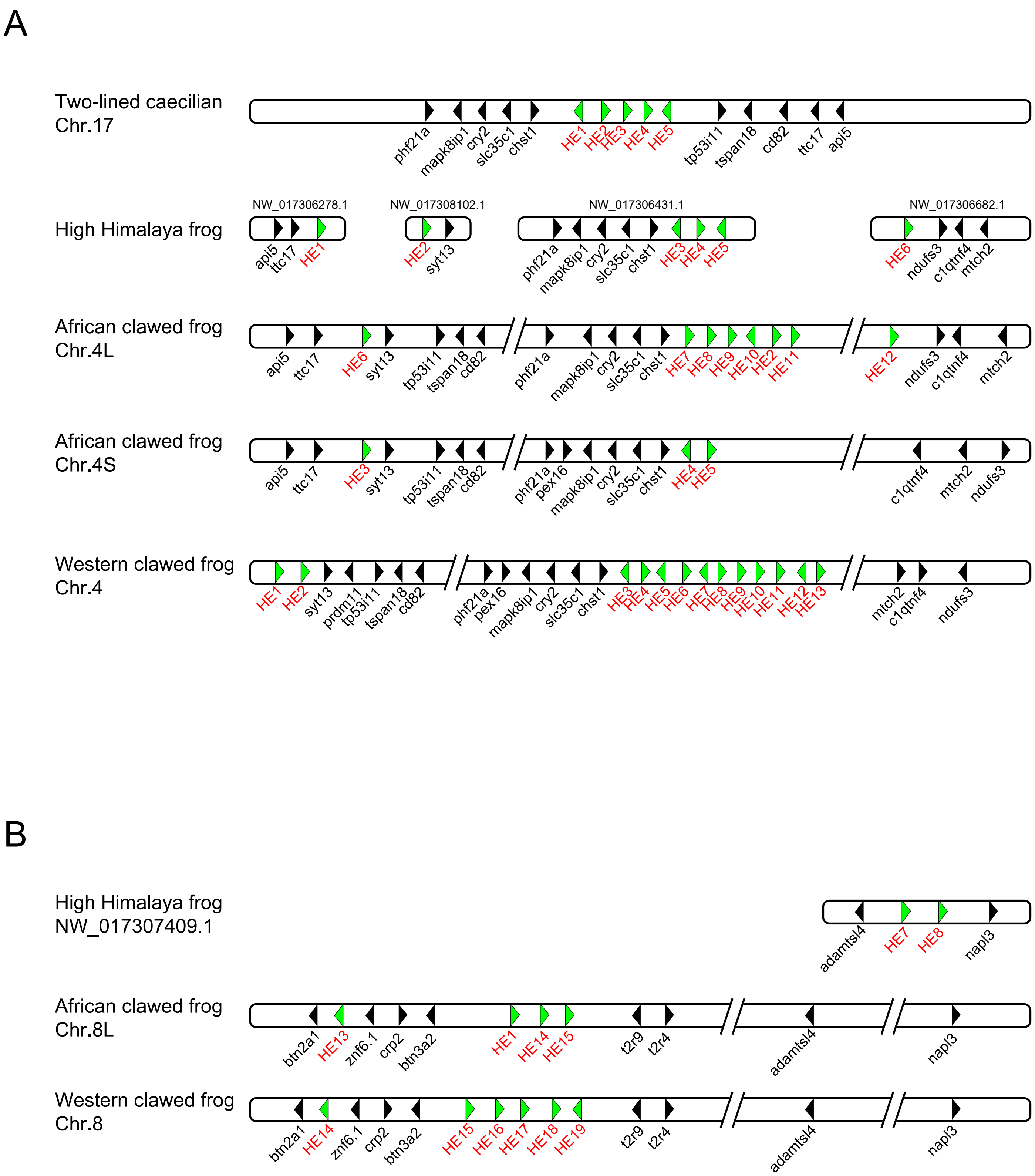
A
Two-lined caecilian
Chr.17
phf21a mapk8ip1 cry2 slc35c1 chst1 HE1 HE2 HE3 HE4 HE5 tp53i11 tspan18 cd82 ttc17 api5
High Himalaya frog
NW_017306278.1
api5 ttc17 HE1
NW_017308102.1
HE2 syt13
NW_017306431.1
phf21a mapk8ip1 cry2 slc35c1 chst1 HE3 HE4 HE5
NW_017306682.1
HE6 ndufs3 c1qtnf4 mtch2
African clawed frog
Chr.4L
api5 ttc17 HE6 syt13 tp53i11 tspan18 cd82 phf21a mapk8ip1 cry2 slc35c1 chst1 HE7 HE8 HE9 HE10 HE2 HE11 HE12 ndufs3 c1qtnf4 mtch2
African clawed frog
Chr.4S
api5 ttc17 HE3 syt13 tp53i11 tspan18 cd82 phf21a pex16 mapk8ip1 cry2 slc35c1 chst1 HE4 HE5 c1qtnf4 mtch2 ndufs3
Western clawed frog
Chr.4
HE1 HE2 syt13 prdm11 tp53i11 tspan18 cd82 phf21a pex16 mapk8ip1 cry2 slc35c1 chst1 HE3 HE4 HE5 HE6 HE7 HE8 HE9 HE10 HE11 HE12 HE13 mtch2 c1qtnf4 ndufs3
B
High Himalaya frog
NW_017307409.1
adamtsl4 HE7 HE8 napl3
African clawed frog
Chr.8L
btn2a1 HE13 znf6.1 crp2 btn3a2 HE1 HE14 HE15 t2r9 t2r4 adamtsl4 napl3
Western clawed frog
Chr.8
btn2a1 HE14 znf6.1 crp2 btn3a2 HE15 HE16 HE17 HE18 HE19 t2r9 t2r4 adamtsl4 napl3

Fig. S5

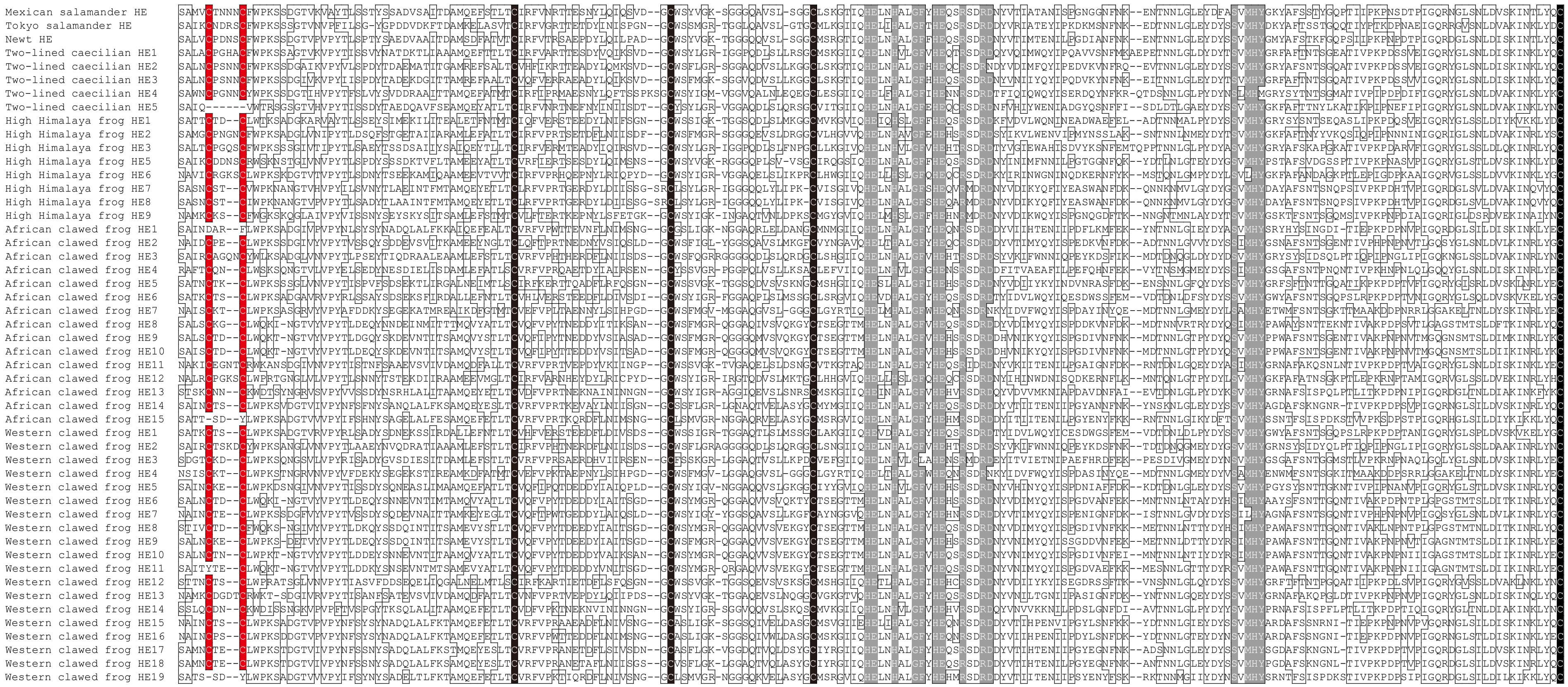

Fig. S6
